# In Vitro Self-Circularization Methods Based on Self-Splicing Ribozyme

**DOI:** 10.3390/ijms25179437

**Published:** 2024-08-30

**Authors:** Kyung Hyun Lee, Nan-Ee Lee, Seong-Wook Lee

**Affiliations:** 1 R&D Center, Rznomics Inc., Seongnam 13486, Republic of Korea; nelee@rznomics.com; 2 Department of Bioconvergence Engineering, Research Institute of Advanced Omics, Dankook University, Yongin 16890, Republic of Korea

**Keywords:** circular RNA, self-circularization, self-splicing, RNA vaccine, RNA therapeutics, ribozyme, group I intron, group II intron

## Abstract

In vitro circular RNA (circRNA) preparation methods have been gaining a lot of attention recently as several reports suggest that circRNAs are more stable, with better performances in cells and in vivo, than linear RNAs in various biomedical applications. Self-splicing ribozymes are considered a major in vitro circRNA generation method for biomedical applications due to their simplicity and efficiency in the circularization of the gene of interest. This review summarizes, updates, and discusses the recently developed self-circularization methods based on the self-splicing ribozyme, such as group I and II intron ribozymes, and the pros and cons of each method in preparing circRNA in vitro.

## 1. Introduction

Recently, circular RNA (circRNA) has been considered a promising alternative to linear mRNA for various biomedical applications [1,2], such as the mRNA vaccine [3,4,5,6,7]. Therefore, efficient in vitro circRNA preparation methods are of great interest in this area [8]. Considering the pros and cons of in vitro methods for circRNA preparation, self-splicing ribozyme-based self-circularization methods would be the most simple and efficient and thus more practical for commercial and biomedical applications than chemical- and ligase-based methods [9,10,11].

As a representative self-splicing ribozyme-based method developed about 30 years ago [12], the permuted intron–exon (PIE) method using group I introns has been improved by rationally designing the construct [2,13,14]. Although the PIE method can provide circRNAs in vitro with good efficiency for gene of interest (GOI) that is not too long, it has intrinsic issues to overcome. For example, it generally leaves extraneous sequences such as exon 1/exon 2 (E1/E2) fragments, two sequences corresponding to exon fragments of the PIE construct, which are required to determine the splicing sites [12,13] or spacer in the generated circRNA, which may trigger unwanted innate immune responses in cells [15]. Those remnant fragments probably tend to form stable RNA duplexes, which may lead to unwanted immune responses. Therefore, improved versions of the group-I-intron-based PIE method [16], a group-II-intron-ribozyme-based PIE approach [17], and a new group-I-intron-ribozyme-based strategy [18,19] have been designed to overcome shortcomings of the conventional PIE method.

This review introduces recent updates on in vitro self-circularization methods based on the self-splicing ribozyme to overcome the conventional PIE method’s shortcomings. It then discusses the advantages and disadvantages of these methods, as well as possible future directions in terms of efficient in vitro circRNA production and engineering.

## 2. Group I Intron PIE-Based Methods

**Figure 1:**
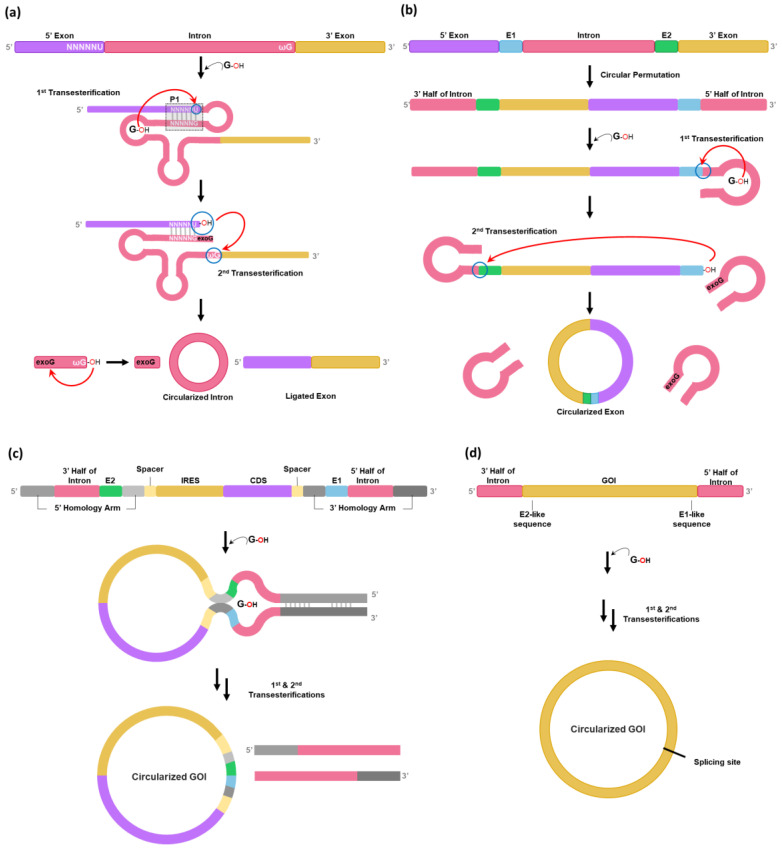
PIE-based in vitro self-circularization methods. (**a**) Example of self-splicing reaction by a *Tetrahymena* group I intron ribozyme. The self-splicing reaction proceeds via two consecutive transesterifications, resulting in excision of the group I intron and ligation of flanking exons. After P1 helix formation between 5′-NNNNNU-3′ and 5′-GN′N′N′N′N′-3′ (internal guide sequences), the 5′ splice site is attacked by the 3′ hydroxyl group of the exogenous guanosine (exoG) within the catalytic core of the group I intron (first transesterification). Subsequently, the 3′ splice site is attacked by the generated 3′-OH group on the uridine at the 3′ terminus of the 5′ exon (second transesterification). As a result, the ligated exon and the circularized intron circularized by a nucleophilic attack of the terminal guanosine (ωG) in the group I intron can be generated. (**b**) In the PIE method, circularly permuted forms of the group I introns interrupted by exons can fold into active conformations. As first transesterification, exoG attacks the 5′ splice site at the 3′ termini of E1; then, the generated 3′-OH group on the uridine at the 3′ termini of E1 attacks the 3′ splice site at the 5′ termini of E2 as a second transesterification. After two transesterification reactions via the normal self-splicing pathway, as described, a circular exon instead of a circular intron can be generated. (**c**) The PIE method, improved by introducing homology arms and spacers, can form an isolated splicing bubble. The two consecutive transesterification reactions, as described in Figure 1b, can be increased in the isolated splicing bubble, and the reaction leaves extraneous sequences, including E1/E2 fragments, in the generated circRNA. (**d**) Splice sites can be concealed in GOI (IRES or CDS region) via the Clean-PIE strategy. The splicing site can be selected from any region of the GOI if there are E1/E2-like sequences.

It is known that a circular intron can be generated as a by-product after two consecutive transesterification reactions for the self-splicing reaction of the group I intron precursor RNAs [20] (Figure 1a). To generate a circular exon instead of a circular intron, the PIE method was developed in 1992 from the hypothesis that circularly permuted forms of the group I intron interrupted by an exon could fold into an active conformation for self-splicing to generate the circular form of an exon [12] (Figure 1b). However, the original PIE method has only been applied for the self-circularization of shorter RNAs such as short exon sequences (100 nt) [21] or aptamers [22] as longer GOI between splice sites might reduce the ability of splice sites to interact with one another, thus reducing the self-circularization efficiency [2].

Therefore, the PIE method has been optimized by rationally designing the PIE construct to be used for longer GOIs [2,13,14]. In 2018, Wesselhoeft et al. optimized the PIE reaction using group I introns present in the thymidylate synthase (Td) gene of bacteriophage T4 or in *Anabaena* pre-tRNA for longer RNA circularization by introducing homology arms and spacer sequences [2] (Figure 1c). Homology arms placed at the 5′ and 3′ ends of the precursor RNA are able to bring the 5′ and 3′ splice sites into proximity with one another, resulting in the increased self-splicing efficiency for longer GOIs [2]. Spacer sequences can also improve circularization efficiency by separating the IRES (internal ribosome entry site) and the 3′ PIE splice site for the correct folding of the group I intron [2]. The formation of a sheltered splicing bubble can also be promoted by introducing homology arms in the spacer regions (Figure 1c), which can increase self-circularization efficiency. Although self-circularization can be completed during in vitro transcription (IVT), an additional reaction (55 °C incubation for 15 min with 2 mM GTP) after DNase treatment is required in some cases. Notably, utilization of the *Anabaena* catalytic intron resulted in a 37% reduction in circRNA nicking compared to the T4 catalytic intron [2]. Nicking is a common issue during circRNA production and purification.

However, the PIE method generally leaves extraneous sequences, such as E1/E2 fragments essential for catalytic activity, which determine the 5′ and 3′ splice sites via the interaction between the internal guide sequence (IGS) and the neighboring loop structure [23] or spacer sequences in the circRNA, which can form stable intramolecular RNA duplexes and may trigger unwanted innate immune responses in cells [15]. Furthermore, obligatory incorporation of E1/E2 and spacer sequences are not sufficient to generate small circRNAs. In addition, if circRNAs are required to have exactly the same sequences as their native ones, incorporation of E1/E2 and spacer sequences into GOI is not appropriate as the generated circRNAs will differ from their native sequences [19].

To avoid an issue of leaving extraneous sequences, Qiu et al. have developed a Clean-PIE method using the Td group I intron of the T4 phage [16] (Figure 1d). In this strategy, minimal-sized E1/E2 fragments-like sequences are screened from GOI sequences, as Rausch et al. have reported that the self-circularization efficiency of the Td group I intron can be affected by variations in the E1 and E2 sequences generated by serial mutagenesis and in vitro selection studies [23], suggesting that sequence restrictions, such as E1/E2 requirement, can be circumvented. In other words, sequences/structures that function like E1/E2 can be concealed in open reading frame (ORF) or in IRES instead of using natural E1/E2 sequences in the exon.

As a previous screening strategy to select E1/E2-like sequences [23] was inefficient for self-circularization of larger circRNAs, a design principle was further improved for the Clean-PIE method [16]. Qiu et al. have found that UUGGGUCU (TTGGGTCT in DNA), which can form P1 and part of P9 with IGS in the Td group I intron, are the most effective E1/E2 sequences for the PIE method [16]. Thus, these short sequences can be concealed in the GOI region by modifying codon sequences without changing amino acid sequences. For example, GATGGATCA sequences (codons for Asp, Gly, and Ser) in the coding sequence (CDS) region of the EGFP gene can be changed to GATGGGTCT (codons for Asp, Gly, and Ser), which are almost identical to the ideal TTGGGTCT sequences. To design the Clean-PIE construct efficiently, Qiu et al. have developed an algorithm for assisting the nucleotide-replacement method with a scoring system to optimize potential E1/E2 fragments in the GOI region [16].

Clean-PIE strategy would be ideal for group I introns such as the Td group I intron, which has relatively short E1/E2 fragments, for base pairing with IGS for catalytic activity due to the ease of prediction/screening of E1/E2-like sequences [23]. As 99.9% of genes with sizes > 500 bp contain at least one E1/E2-like segment scoring 13 points (score ranges from 0 to 16, with a higher score predicting higher circularization efficiency), the strategy is generally applicable for circularization of most GOIs [16]. In addition, 82.9% of *Homo sapiens* genes with a size > 1000 bp contain more than one ideal TTGGGTCT sequence with the highest efficiency [16].

## 3. Group II Intron PIE-Based Methods

**Figure 2:**
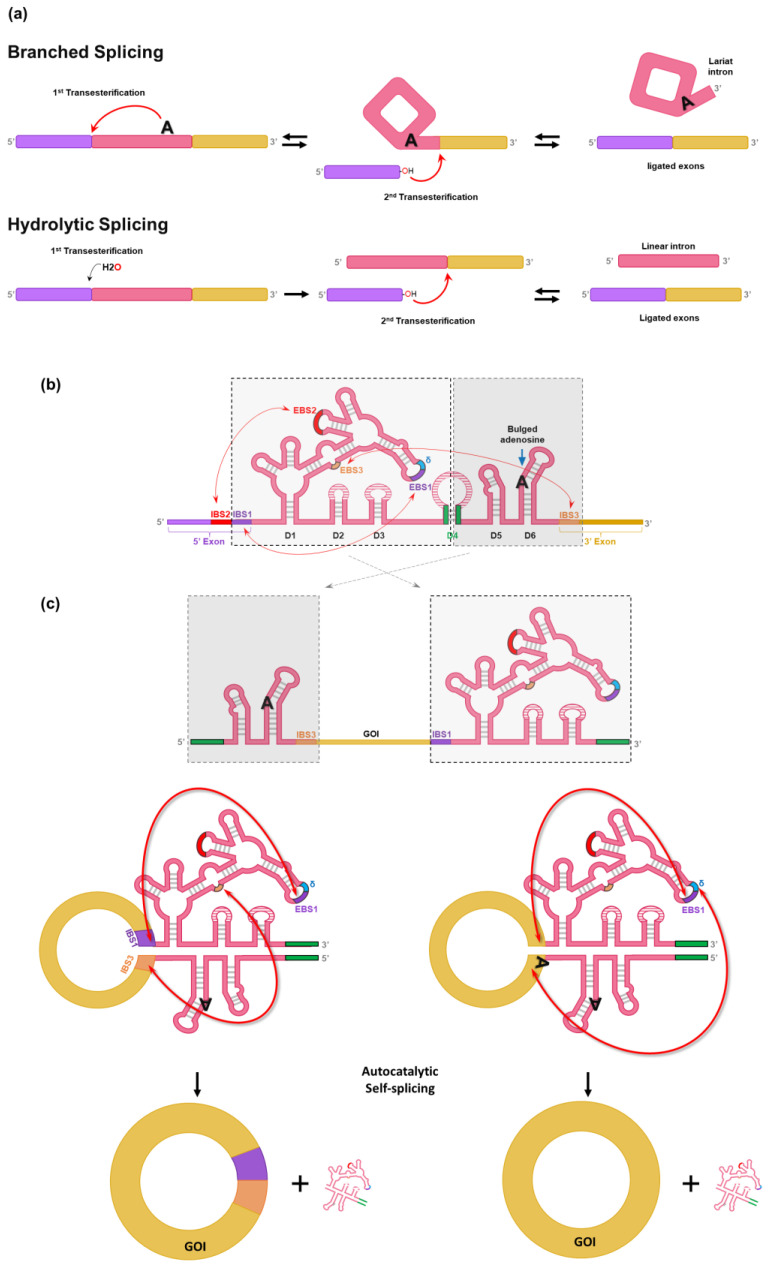
Group II intron self-splicing reaction and group-II-intron-based PIE method for in vitro self-circularization. (**a**) Branched or hydrolytic self-splicing reaction by the group II intron. In the branched splicing pathway, the nucleophile for the first transesterification is a specific bulged adenosine within the group II intron; whereas in the hydrolytic splicing pathway, the nucleophile for the first step is a water molecule or hydroxide ion. The 3′-OH terminus of the 5′ exon generated by the first transesterification attacks and connects with the 3′ splice site (second transesterification). In the branched splicing pathway, a lariat intron is generated, but in the hydrolytic splicing pathway, a linear intron is released. (**b**) Detailed secondary structure and self-splicing reaction of the group II intron. Each arrow indicates the interaction between the exon binding site (EBS) and the intron binding site (IBS). As described in Figure 2a, a bulged adenosine in D6 attacks the 5′ splice site determined by EBS1 and IBS1 interaction (first transesterification); then, the generated 3′-OH terminus of the 5′ exon attacks the 3′ splice site determined via EBS3 and IBS3 interaction (second transesterification). (**c**) Schematic diagram for the design of the group-II-intron-based PIE (CirCode) system. The group II intron for self-splicing was split into two fragments at the D4 domain and permuted, and GOI was inserted between the split introns. Initial design of the group II-based PIE system which generates circRNA with a short intronic scar of 12 nt (IBS1 and IBS3) (Left). Design for scarless circRNA generation using group-II-intron-based PIE system (Right). The modifications of intronic sequences result in the specific recognition of the splicing junctions in GOI. Thus, a scarless circRNA can be generated by self-circularization.

As an alternative strategy, the group II intron of *Clostridium tetani* [24], with structure engineering, can be used instead of using the group I intron in PIE-based method [25] to generate scarless circRNA [17]. Generally, self-splicing by the group II intron occurs through one of two pathways in parallel (branched splicing or hydrolytic splicing) (Figure 2a) [26,27,28]. In the branched splicing pathway, the 2′-OH group of the intron branchpoint (bulged adenosine) performs the nucleophilic attack on the 5′ splice site (first transesterification), which yields a free 5′ exon and a lariat-3′ exon intermediate. Then, the free 5′ exon attack the 3′ splice site (second transesterification), resulting in the formation of ligated exons and lariat intron. Both transesterifications are reversible, enabling reverse splicing that allows group II introns to reverse-splice into new target sites [26,27,28]. In the hydrolytic splicing pathway, a water molecule or hydroxide ion works as a nucleophile for the first transesterification. The second transesterification occurs the same as in branched splicing reaction, but the products are ligated exons and a linear intron, instead of lariat intron [26,27,28].

Group II introns are characterized by conserved secondary structures and have six structural domains (D1 to D6) (Figure 2b). Although group II introns have similar overall secondary structures, three major groups (IIA, IIB, and IIC) distinguished by specific variations in peripheral structures are critical for self-splicing reaction [26,27].

D1 contains several short exon binding sites (EBS) to determine the specificity of self-splicing (Figure 2b) [26,27]. Specifically, the EBS1 in D1 binds with the intron binding site (IBS1) to determine the 5′ splicing junction. Moreover, the interaction between IBS3 and EBS3 (for IIB and IIC introns) or the δ base (for IIA intron) in D1 determines the 3′ splicing junction. D2 and D3 stabilize interactions between other domains and increase the catalytic activity for self-splicing. D4 is not essential for self-splicing activity, but it contains ORF encoding an intron-encoded protein (IEP) known as a maturase, which is required for in vivo reaction. In contrast, group II self-splicing requires only the correct folding of the ribozyme structure and Mg^2+^ for the in vitro reaction [28]. The highly conserved D5 interacts with D1 to form the catalytic core that binds two divalent metal ions essential for self-splicing reaction [28]. D6 harbors the bulged adenosine nucleotide that acts as a nucleophile in the first transesterification reaction for self-splicing [28].

Chen et al. have designed a split-intron system (CirCode system) which contains GOI flanked by the two half-introns, like PIE (Figure 2b,c) [17]. To generate circRNA without extraneous sequences (12 nt for group II intron of *C. tetani*), exon binding sites were engineered so that scarless circRNA could be generated in vitro [17]. At first, group II intron of *C. tetani* was split like the PIE method (Figure 2c). The stem region of D4 was separated and placed into each end of the designed RNA construct, thus forming a stem structure via complementary binding that helps the folding of the active conformation of the group II intron (green lines in Figure 2c). However, the initial design of group-II-intron-based PIE still leaves a short scar of 12 nt (6 nt sequences of IBS1 and IBS3) (Figure 2c, left).

The design was further modified to generate scarless circRNA by changing the EBSs in D1. As a result, EBS1 and sequence upstream of EBS1 can form base pairs with the 3′ and 5′ end of GOI, respectively (Figure 2c, right). Therefore, the CircCode system enabled the generation of circRNA without an intronic scar [17].

Self-circularization can be observed during IVT, and the purified RNAs can be further circularized by additional reaction as an option (incubation at 53 °C in the presence of 50 mM Tris-HCl at pH 7.5, 50–100 mM NaCl, and 10–20 mM MgCl_2_ after 5 min heating at 75 °C and cooling down to 45 °C) [17].

However, due to the close proximity of intron structure and IRES structure in PIE-based system, spacer sequences are still required because how far apart the two structures are can affect circularization efficiency [2,17]. In addition, the formation of 2′,5′-phosphodiester bonds at the ligation site could be an issue, although this remains controversial as the precise mechanism is unclear [9,11,29,30].

## 4. Group I intron Self-Targeting and Splicing-Based Methods

Recently, two research groups, including our team, have reported self-targeting and splicing (STS)-based self-circularization methods based on the *Tetrahymena* group I intron ribozyme [18,19].

**Figure 3:**
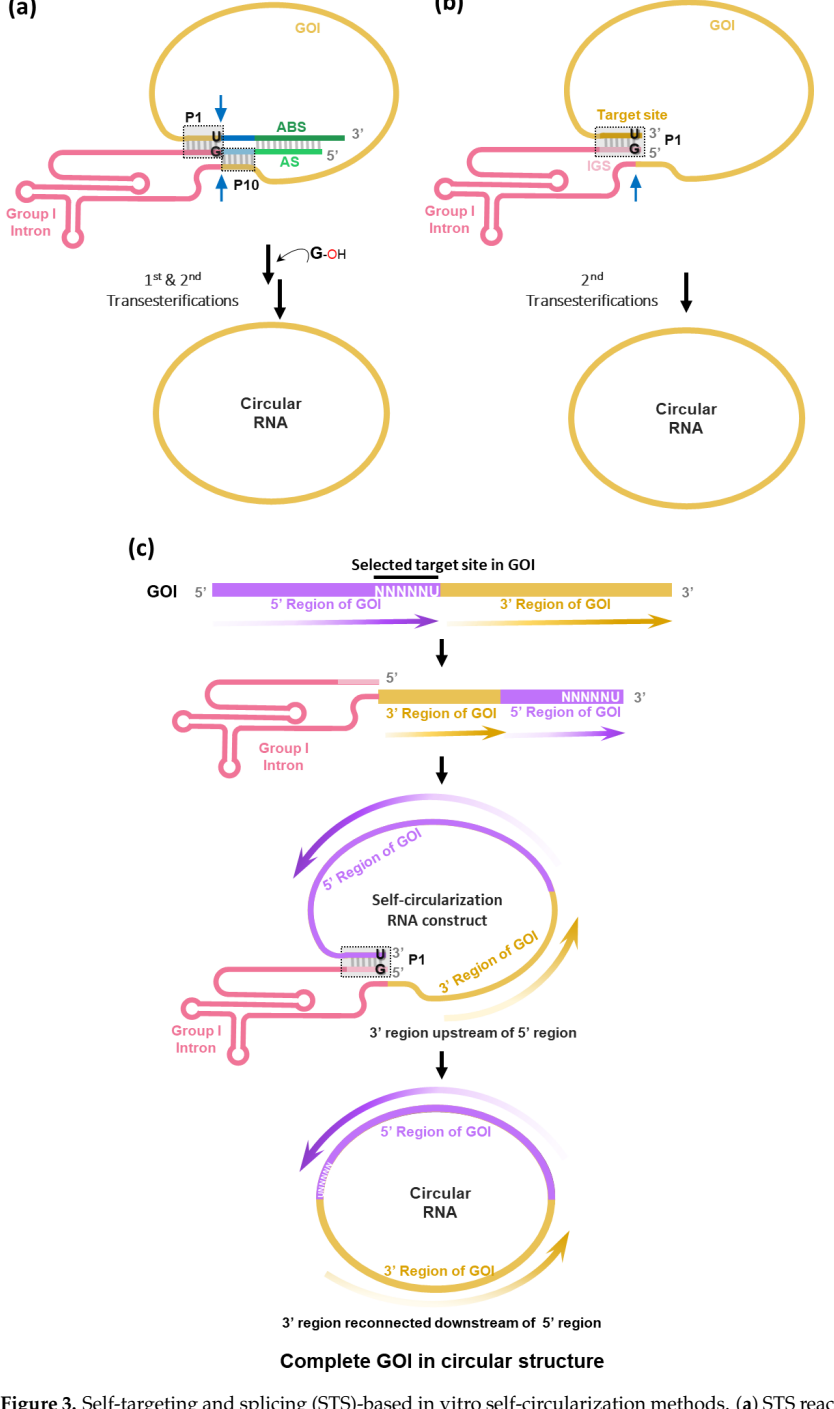
Self-targeting and splicing (STS)-based in vitro self-circularization methods. (**a**) STS reaction by the self-circularization RNA construct, which consists of P1, P10 helixes in the group I intron, and AS (antisense sequence)/ABS (antisense binding sequence) components, which could affect the specificity and efficacy of splicing reaction. The reaction occurs via two consecutive transesterification reactions identical to reactions during self-splicing, as shown in Figure 1a. Briefly, the target site (5′-NNNNNU-3′) in the GOI is paired by IGS (5′-GN′N′N′N′N′-3′), forming a P1 helix structure. P1 and P10 helixes define the 5′ and 3′ splice sites. For the first transesterification, the 3′-OH group of exogenous guanosine attacks the uridine at the 3′ end of the target site. Then, ωG at the 3′ end region of the group I intron is attacked by the 3′-OH generated at the end of the cleaved target site (second transesterification), resulting in the generation of circRNA via a ligation. As the group I intron and related components are released after reactions, the generated circRNAs do not harbor any extraneous sequences. (**b**) End-to-end STS reaction via a simple P1 RNA construct. Only the second transesterification reaction in the normal pathway is required for the self-circularization. In this case, the first transesterification described in Figure 3a can be skipped as the 3′-OH of the target site is already exposed at the end of the target site in this design. Only the second transesterification occurs, resulting in the efficient generation of circRNA. (**c**) Any position in GOI can be selected as a target site for self-circularization. Split GOI parts can be reconnected in the correct order after self-circularization.

To generate circRNA without extraneous sequences (i.e., intronic scars) such as E1/E2 fragments of the PIE construct, we have rationally designed an RNA construct using the *Tetrahymena* group I intron trans-splicing ribozyme [31] to enable self-circularization through end-to-end STS reaction [18] (Figure 3a). In this strategy, the STS reaction cleaves the 3′ end of GOI and then links the 5′ end of GOI, which generates scarless circRNAs and releases group I intron ribozyme. Of note, we found that only the simple P1 helix structure formation (complementary base pairing between target site with 5′-NNNNNU-3′ and IGS with 5′-GN′N′N′N′N′-3′) at the 3′ and 5′ ends of the self-circularizable group I intron RNA construct was sufficient to generate circRNA in vitro (Figure 3b). In the report by Cui et al. [19], only the full construct with antisense interaction was used for self-circularization (Figure 3a).

In terms of circRNA engineering, any sequences with 5′-NNNNNU-3′—preferably AU-rich sequences—in GOI can be targeted if only one specific target sequence is present in the GOI. When the target site is selected, the GOI region following the target site can be sent to the next position of the group I intron in the self-circularization construct. Therefore, after self-circularization, GOI regions can be reconnected with each other, generating the circRNA with the complete GOI in the correct order (Figure 3c).

Self-circularization can be completed during IVT efficiently without additional reaction [2,7] (additional reaction is optional for the STS reaction [19]). Its efficiency is comparable to that of the PIE method. As the STS-based method avoids leaving unwanted sequences, Cui et al. have perfectly mimicked the native sequence of circRNA, and the prepared circRNAs performed their original biological functions in cell-based assays [19]. In this study, circRNA expression vector was used for cell-based assays instead of directly using in vitro self-circularized circRNAs. Regardless of using in vitro-prepared circRNAs [18] or circRNA expression vector system [19], higher protein expression levels, as well as longer-lasting expression, were observed compared to the use of linear RNAs in cells [18]. These results are consistent with previous reports [2,13] using circRNAs prepared by the PIE method.

## 5. Discussions

Recently, practical self-splicing ribozyme-based in vitro circRNA preparation methods have been receiving great attention. Here, we reviewed recent updates on those methods such as PIE-based and STS-based methods. Based on our opinions on each method and related reports, the pros and cons of each method are summarized in Table 1.

Generally, the PIE method using the group I intron has been widely used to prepare circRNA in vitro. However, the conventional PIE method generally leaves extraneous sequences such as E1/E2 fragments and spacer sequences in the generated circRNAs. For example, in the circRNAs generated by the *Anabaena* group-I-intron-based PIE, scar sequences consist of 62 nt of intronic scar (15 nt of E1 plus 52 nt of E2) and 108 nt of spacers (69 nt and 39 nt of two spacer regions) with internal homology arms, which can limit the flexibility of design/engineering for circRNAs and may introduce some unwanted side effects [19], such as an innate immune response [15]. However, observation of unwanted innate immunity via an intronic scar is still controversial as circRNA generated via the PIE method showed a negligible level of innate immune responses in Wesselhoeft’s work [13]. The possibility of different levels of impurity after circRNA purification in each report cannot be excluded [13,15].

With the Clean-PIE method, sequences/structures that function like E1/E2 can be concealed in the GOI region. Although 99.9% of the genes with sizes > 500 bp contain at least one E1/E2-like sequences with a prediction of good efficiency [16], genes with sizes < 500 bp do not. The optimized codon usage in the original CDS should also be changed to use better E1/E2-like function, if required. This method has not yet been validated by a peer-reviewed journal.

PIE using the group II intron (CirCode system) avoids leaving E1/E2 fragments in the generated circRNAs. However, PIE-based methods require spacer sequences between IRES and intron structures as the close proximity of the two structures can affect circularization efficacy [2,17]. Moreover, the exon binding site in the D1 domain of the group II intron that was modified to bind to the GOI for scarless circRNA generation is not universally applicable as the GOI sequences are different for each GOI. Therefore, the D1 sequence should be modified for each specific GOI [32]. This method has not yet been validated by a peer-reviewed journal. There is another CirCode system with the same name, reported in a peer-reviewed journal [33], but this system is about a software tool designed to identify circRNAs with translation potential.

The STS method, which has a distinct mechanism from PIE, avoids leaving unwanted sequences/structures in the generated circRNA. Ideally, even spacer sequences are not required as a target site can be selected to separate IRES and ribozyme structures in the designed self-circularization RNA construct (K.H. Lee and S.-W. Lee, unpublished data) [34]. However, the introduction of spacers such as polyAC could improve the translation efficiency probably due to the binding of some intracellular factors such as polyA binding proteins [14,16].

With the STS method, P1/P10 and AS/ABS can be optimized to increase the self-circularization efficiency [18]. In addition, as a target site also affects the self-circularization efficiency, a target site with good self-circularization efficiency can be selected to design the optimal self-circularization construct. Therefore, in addition to the strategy of using AU-rich target sites [18], an efficient target site selection strategy needs to be established to reduce the laborious design/test cycles.

Self-circularization based on self-splicing ribozyme-based methods can be completed during IVT, although additional incubation reaction would increase the self-circularization efficiency, suggesting that an extra ligation reaction using ligase is not needed for the circularization. Therefore, circRNA production yields would be proportional to the IVT scale, suggesting that these methods would be more practical than other in vitro circRNA preparation methods.

However, there are still some shortcomings for self-splicing ribozyme-based in vitro circRNA preparation methods. For example, the appropriate purification methods for large-scale production with higher purity and negligible nick formation should be validated.

Both PIE- or STS-based methods are expected to be further optimized rapidly to overcome their shortcomings for various biomedical applications with unmet needs.

## Figures and Tables

**Table 1:** Pros and cons of self-splicing ribozyme-based self-circularization methods.

Method	Pros	Cons
Group-I-intron-based PIE	Well established	Extraneous sequences (E1/E2 and spacer) Limited engineering/design flexibility due to the scar sequence
Group-I-intron-based Clean-PIE	No intronic scar (E1/E2-like sequences/structures in GOI)	Some GOIs (e.g., shorter GOIs) may not have highly efficient E1/E2-like sequences Codon usage should be changed Not validated by a peer-reviewed journal
Group-II-intron-based PIE	No intronic scar	Engineering of D1 domain for each GOI A 2′-5′-phosphodiester bond at ligation site (controversial) Not validated by a peer-reviewed journal
Group-I-intron-based STS	No extraneous sequences Multiple options of optimization/engineering	Need to be further established (e.g., target site selection strategy to choose a target site with high self-circularization efficacy for various GOIs, etc.)
